# Development and Feasibility of a Mobile Health–Supported Comprehensive Intervention Model (CIMmH) for Improving the Quality of Life of Patients With Esophageal Cancer After Esophagectomy: Prospective, Single-Arm, Nonrandomized Pilot Study

**DOI:** 10.2196/18946

**Published:** 2020-08-18

**Authors:** Chao Cheng, Rainbow Tin Hung Ho, Yan Guo, Mengting Zhu, Weixiong Yang, Yiran Li, Zhenguo Liu, Shuyu Zhuo, Qi Liang, Zhenghong Chen, Yu Zeng, Jiali Yang, Zhanfei Zhang, Xu Zhang, Aliza Monroe-Wise, Sai-Ching Yeung

**Affiliations:** 1 Department of Thoracic Surgery The First Affiliated Hospital of Sun Yat-sen University Guangzhou China; 2 Department of Social Work & Social Administration The University of Hong Kong Hong Kong China; 3 Centre on Behavioral Health The University of Hong Kong Hong Kong China; 4 Department of Medical statistics School of Public Health Sun-Yat-Sen University Guangzhou China; 5 Sun Yat-sen Center for Migrant Health Policy Guangzhou China; 6 Sun Yat-sen Center for Global Health Guangzhou China; 7 Department of Clinical Nutrition The First Affiliated Hospital of Sun Yat-sen University Guangzhou China; 8 Department of Rehabilitation Medicine The First Affiliated Hospital of Sun Yat-sen University Guangzhou China; 9 Department of Thoracic Surgery Sun Yat-Sen University Cancer Center Guangzhou China; 10 Department of Global Health University of Washington Seattle, WA United States; 11 Department of Emergency Medicine Division of Internal Medicine The University of Texas MD Anderson Cancer Center Houston, TX United States

**Keywords:** esophageal cancer, quality of life, nutrition, physical exercise, psychological support, mobile health, mHealth

## Abstract

**Background:**

Patients with esophageal cancer often experience clinically relevant deterioration of quality of life (QOL) after esophagectomy owing to malnutrition, lack of physical exercise, and psychological symptoms.

**Objective:**

This study aimed to evaluate the feasibility, safety, and efficacy of a comprehensive intervention model using a mobile health system (CIMmH) in patients with esophageal cancer after esophagectomy.

**Methods:**

Twenty patients with esophageal cancer undergoing the modified McKeown surgical procedure were invited to join the CIMmH program with both online and offline components for 12 weeks. The participants were assessed before surgery and again at 1 and 3 months after esophagectomy. QOL, depressive symptoms, anxiety, stress, nutrition, and physical fitness were measured.

**Results:**

Of the 20 patients, 16 (80%) completed the program. One month after esophagectomy, patients showed significant deterioration in overall QOL (*P*=.02), eating (*P*=.005), reflux (*P*=.04), and trouble with talking (*P*<.001). At the 3-month follow-up, except for pain (*P*=.02), difficulty with eating (*P*=.03), dry mouth (*P*=.04), and trouble with talking (*P*=.003), all other QOL dimensions returned to the preoperative level. There were significant reductions in weight (*P*<.001) and BMI (*P*=.02) throughout the study, and no significant changes were observed for physical fitness measured by change in the 6-minute walk distance between baseline and the 1-month follow-up (*P*=.22) or between baseline and the 3-month follow-up (*P*=.52). Depressive symptoms significantly increased 1 month after surgery (*P*<.001), while other psychological measures did not show relevant changes. Although there were declines in many measures 1 month after surgery, these were much improved at the 3-month follow-up, and the recovery was more profound and faster than with traditional rehabilitation programs.

**Conclusions:**

The CIMmH was feasible and safe and demonstrated encouraging efficacy testing with a control group for enhancing recovery after surgery among patients with esophageal cancer in China.

**Trial Registration:**

Chinese Clinical Trial Registry (ChiCTR-IPR-1800019900); http://www.chictr.org.cn/showprojen.aspx?proj=32811.

## Introduction

Esophageal cancer is the third most common cancer and the fourth most common cause of cancer death in China [1]. Esophagectomy is the major curative treatment option and is often performed in combination with neoadjuvant chemotherapy or chemoradiotherapy [2]. The surgery is considered extensive and entails a more than 40% risk of postoperative complications [3,4]. For example, anastomotic leakage is one of the most common postoperative complications, and it occurs in 5%-20% of patients with esophageal cancer [5,6]. Complications such as this may increase hospital stay, delay oral feeding, lead to malnutrition, increase psychological burden, cause poor quality of life (QOL), and subsequently worsen the long-term survival of patients [7-10].

Enhanced recovery after surgery (ERAS) is a patient-centered, evidence-based multimodal and multidisciplinary approach for promoting early recovery and reducing complications among patients after surgery [11]. Previous studies have shown the beneficial effects of interventions based on ERAS guidelines to improve the nutrition and physical status of patients with head, neck, and breast cancer after surgery [12,13]. For patients with esophageal cancer, existing interventions may be effective, but each intervention program usually focuses on one specific aspect of health such as nutrition or exercise [14-16]. To meet different needs, such as overcome postoperative complications and malnutrition, patients need to meet different professionals and to return to the hospital frequently for different appointments, creating potential obstacles for those who have been discharged from the hospital, especially those residing in rural or remote areas. Moreover, individual intervention programs may lack coherence. A comprehensive intervention program tailored to individual patients and designed to support patients holistically in all aspects, including nutrition, physical exercise, and psychosocial support, is thus urgently warranted for patients with esophageal cancer after esophagectomy.

In addition, effective information delivery and adherence to follow-up with health care professionals are of high priority in cancer care and are key elements of successful implementation of ERAS. In the past, most interventions were delivered face-to-face in either individual or group settings [12,17]. However, a face-to-face approach may not be easy for patients who live far away from the hospital or those who have physical difficulties in travelling. Therefore, a home-based supportive care intervention is warranted for discharged patients with esophageal cancer after esophagectomy [18,19]. An offer of timely guidance of home-based supportive care to patients can help reduce symptom distress or anxiety and prevent complications, and thus promote physical rehabilitation after surgery. In recent years, the wide adoption of mobile technology (eg, smartphones and mobile apps) offers a promising platform for efficient and accessible intervention delivery [20]. Mobile health (mHealth) refers to health care or health-related services delivered by mobile or other wireless devices, such as smartphones and tablets [21,22]. Several studies have demonstrated the efficacy of mHealth interventions for improving overall QOL in patients with endometrial, breast, or lung cancer [23-25]. However, there has been no mHealth-based intervention to improve QOL in patients with esophageal cancer after esophagectomy. Using an mHealth system on a mobile platform may be ideal for patients who have difficulty in making frequent follow-up visits to a hospital, which can be particularly challenging in China as hospitals are always situated in city centers. As 98.5% of people aged 50 to 80 years use the WeChat platform in China [26], an mHealth program delivered via WeChat would reach a substantial percentage of patients with cancer in China to support them at home.

Therefore, we designed the first comprehensive intervention model supported by mHealth (CIMmH) delivered on the WeChat platform, providing nutrition, exercise, and psychological support for patients with esophageal cancer after esophagectomy. This prospective pilot study aimed to examine the feasibility and safety of a 12-week CIMmH. The study will support the development of future programs for those patients with cancer who may not be able to visit the hospital frequently or who live in rural areas in China.

## Methods

### Study Design

This prospective, single-arm, nonrandomized pilot study was conducted at the First Affiliated Hospital of Sun Yat-sen University in Guangzhou, China, which has 2850 beds serving 4.9 million patients each year. The Department of Thoracic Surgery cares for more than 300 patients with esophageal cancer each year. The study was registered at the Chinese Clinical Trial Registry (ChiCTR-IPR-1800019900) and was approved by the ethics committee of the First Affiliated Hospital of Sun Yat-sen University according to the Declaration of Helsinki.

### Participants

Patients diagnosed with esophageal cancer and scheduled for esophageal radical resection were referred by thoracic oncologists in the inpatient department of the hospital from December 2018 to October 2019. Those who met the eligibility criteria were invited to join the study. The inclusion criteria of the study were a diagnosis of esophageal cancer, suitability for the modified McKeown procedure (thoracoscopic esophageal mobilization three-incision esophagectomy) [27] and jejunostomy before surgery, age between 18 and 75 years with an expected survival of 12 months or longer, normal preoperative gastrointestinal function, Karnofsky performance scores of ≥90 before surgery, ability to walk continuously for 6 minutes or longer before surgery, own WeChat account or an account among family members, cognitive capability to understand Chinese and the study procedures, and ability to provide written informed consent. Individuals were excluded if they had esophageal carcinoma with distant metastases, were unable to engage in physical exercise owing to medical comorbidities, were HIV seropositive, were pregnant or lactating, were unable to finish the questionnaire owing to mental problems or other reasons, did not undergo R_0_ resection, failed to have a jejunostomy feeding tube fitted, or had other serious medical, psychiatric, or cognitive illnesses that would interfere with their participation. Interested participants had appointments with trained research staff to receive further information about the study, signed the informed consent form, and completed the baseline assessment.

### Study Flow

**Figure 1:**
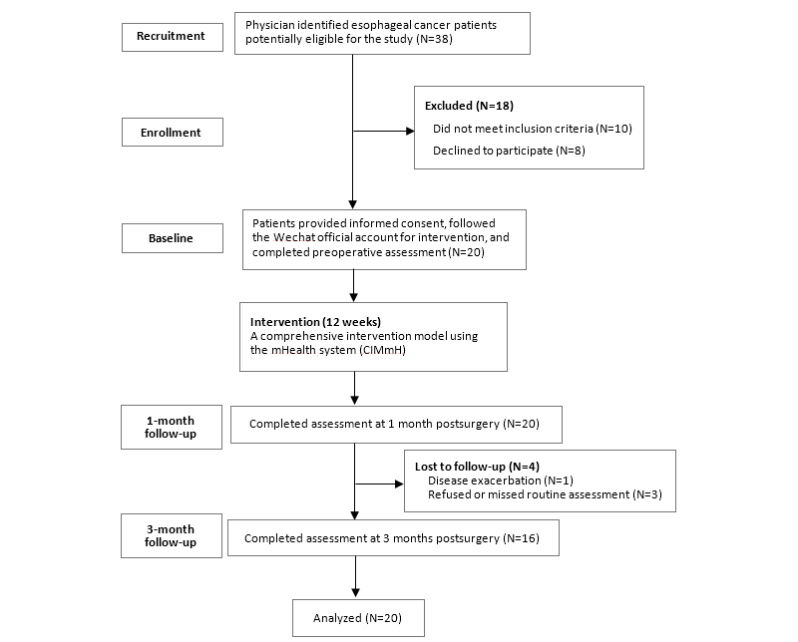
CONSORT flow chart of the study.

Figure 1 depicts the flow chart of the study. Of 38 patients diagnosed with esophageal cancer and scheduled for resection with the modified McKeown surgical approach, 10 did not meet the inclusion criteria and 8 declined to take part in the study. Personal reasons for rejection included concerns for patient privacy from family members and patients’ unstable postoperative condition. Thus, the final sample included 20 patients. The overall response rate was 52.6% (20/38). Of the 20 patients enrolled, 16 (80%) completed the study and 4 (20%) dropped out at the 3-month follow-up owing to disease exacerbation, unwillingness to continue, or missing routine assessments.

### Intervention Protocol

A 3-month CIMmH program was delivered to participants after surgery by specialists in the hospital (offline) and through the enhanced WeChat platform (online). The program included general guidelines on postsurgery recovery, strategies to cope with postoperative complications, nutrition guidelines, physical exercise promotion, and psychological support courses. Details of the CIMmH are provided in Table 1. The components of the CIMmH are presented below.

**Table 1:** Components of the 3-month CIMmH program.

Category	Presurgery	Postsurgery^a^			
	Week 1-2	Week 1 (before discharge)	Week 2-3 (nasogastric tube removed)	Week 4-6 (nasogastric tube feeding tube removed)	Week 7-12
General introduction	Introduction of the CIMmH^b^ program (video by a doctor)	Introduction of the strategies to cope with common postoperative complications	Introduction of the strategies to cope with common postoperative complications	Introduction of the strategies to cope with common postoperative complications	Introduction of the strategies to cope with common postoperative complications
Nutrition	N/A^c^	Standard postoperative nutrition support in the hospital	Rehabilitation guidance of the jejunostomy feeding enteral nutrition period (video by a doctor and nurse) Home jejunostomy feeding enteral nutrition guidance (article) Home enteral nutrition guidance for the use of the feeding tube (video)	Rehabilitation guidance of the transitional period between enteral nutrition and ONS^d^ (video by a doctor and nurse) Home nutrition guidance for the transitional period (article) Nutrition prescription by a nutritionist (face-to-face)	Rehabilitation guidance of ONS and oral intake (video by a doctor and nurse) Home oral nutrition guidance (article) Nutrition prescription by a nutritionist (face-to-face)
Physical exercise	Inspiratory muscle training	Walk promotion	Walk promotion	Walk promotion Baduanjin Qigong (video)	Walk promotion Baduanjin Qigong (video)
Psychological courses	N/A	N/A	N/A	Adapted MBCR^e^ courses (articles and audio)	Adapted MBCR courses (articles and audio)
Data collection	Data collection at baseline (ie, about 1 week before surgery)	N/A	N/A	Data collection at 1 month after surgery	Data collection at 3 months after surgery

^a^Postoperative day (POD) 1: commence 20 mL/h water via jejunostomy feeding; POD 2: commence 20 mL/h jejunostomy feed enteral nutrition suspension; POD 3 to the date of discharge: gradually increase jejunostomy feed to the rate that meets the individual daily energy plan.

^b^CIMmH: comprehensive intervention model using the mobile health system.

^c^N/A: not applicable.

^d^ONS: oral nutrition supply.

^e^MBCR: mindfulness-based cancer recovery.

#### Nutrition Guidelines

Individual nutrition plans were developed by clinical nutritionists and cardiothoracic surgeons based on European Society of Clinical Nutrition and Metabolism guidelines (energy, 30 kcal/kg; protein, 1.5 g/kg ideal body weight) [28]. Patients and/or family caregivers were trained in jejunostomy feeding and used the WeChat platform at home. There were three different periods of nutrition support for patients with esophageal cancer who underwent surgery after discharge as follows: (1) home total enteral nutrition (TEN) on postoperative days (PODs) 8 to 21; (2) partial enteral nutrition (PEN) + oral nutrition supply (ONS) on PODs 22 to 42; and (3) ONS + oral intake on PODs 43 to 90. Detailed individual nutrition plans of each period were delivered by a nutritionist in a face-to-face meeting (offline) at POD 7, POD 21, and POD 42 in the hospital. In addition, three educational readings on general nutrition guidelines for each period (1000 words and 5 minutes of reading on average) and two instructional videos (7 minutes on average) on home-based enteral nutrition were sent to the participants or their caregivers via the WeChat (online) platform.

#### Physical Exercise

The physical exercise protocol consisted of inspiratory muscle training (chest mobilization exercise, flow-oriented incentive spirometry, deep breathing, and coughing exercise), walking exercise [29,30], and Baduanjin qigong [31], which is a mild form of muscular exercise from China involving eight movements. The participants were trained by rehabilitation therapists to perform inspiratory muscle exercises once before surgery lasting about 30 minutes (offline) and were also asked to use these techniques after surgery. Guidance on walking was individually tailored to the participants’ fitness levels measured by the 6-minute walk distance (6MWD) before discharge (around POD 7) and adjusted according to the 6MWD measured at 1 month after discharge.

Baduanjin qigong has been shown to have positive effects on patients with cancer, including alleviating sleep disturbances, strengthening immune function, and improving QOL [32,33]. A video of the Baduanjin qigong exercise was sent to the patients every day from 1 month to 3 months after surgery (online). Patients were encouraged to complete at least one cycle of about 15 minutes every day during this period.

#### Psychological Support

A psychological support program was adapted from mindfulness-based cancer recovery (MBCR) courses [34,35], which have demonstrated effectiveness in reducing stress and depressive symptoms in patients with cancer [36,37]. The adapted MBCR program consisted of four articles on meditation and coping with stress (400 words for each article and average reading time of 3 minutes) and 14 audio clips on meditation and stress reduction (10 minutes on average for each clip). The participants received the program 6 days a week for 2 months from 1 month to 3 months after surgery, with one rest day per week.

#### mHealth Intervention Development

The enhanced WeChat platform was developed by the research team, with three enhanced functions, including automatic intervention delivery, progress monitoring of patient engagement, and personalized feedback with community support.

#### Online Intervention Delivery

Through the enhanced WeChat platform, intervention materials were delivered to the participants and their family caregivers. In order to deliver the targeted intervention at different stages of recovery, the 3-month intervention was divided into the following five stages: (1) enrollment to presurgery; (2) after surgery and before discharge (POD 8); (3) after discharge until before removal of the nasogastric tube (POD 21); (4) before removal of the feeding tube (POD 42); and (5) after removal of the feeding tube until completion (POD 84). Researchers preset the stages for each patient on the online platform, and the corresponding intervention materials for each stage were automatically sent to the users.

#### Progress Monitoring

Patient engagement was tracked and monitored by the enhanced mHealth system, which showed whether the participants had switched on the program and the length of time they stayed on it. In addition, the participants were asked to report their nutrition intake, duration of walking, frequency of practicing Baduanjin qigong exercise, and mood every day on WeChat. The patients received instant and automatic feedback through WeChat and phone calls when needed, to discuss how they had completed the CIMmH program and whether their intake met the nutrition needs.

#### Support Community

An online support community was developed to offer social support through a chat feature. All participants were invited to join, and they were able to post their questions or comments on the WeChat group or via private chat to seek help or share their experiences. Researchers could also respond to messages instantly.

### Data Collection and Measures

The participants were assessed at the following three time points: baseline (about 1 week before surgery) and 1 month and 3 months after surgery. The assessments were conducted in the hospital with the use of tablets and were assisted by trained research staff. Sociodemographic characteristics were collected, and they included age, gender, marital status, education, employment, and income. For measuring patient QOL, the European Organization for Research and Treatment of Cancer-Quality of life Question-Core (EORTC-QLQ-C30, version 3.0) and Oesophageal Cancer Module (EORTC-QLQ-OES-18) questionnaires [38] were used. EORTC-QLQ-C30 is a 30-item measure that includes an overall QOL scale, five functional scales (physical, role, emotional, cognitive, and social), three symptom subscales (fatigue, nausea & vomiting, and pain), and six single items (dyspnea, insomnia, appetite loss, constipation, diarrhea, and financial difficulties). The scores of EORTC-QLQ-C30 range from 0 to 100, with higher scores indicating a higher QOL. The Chinese version of the scale has been used in Chinese patients with cancer and has high validity and reliability [39].

EORTC-QLQ-OES-18 is a supplement of the disease-specific module for patients with esophageal cancer [40]. It consists of four symptom subscales (dysphagia, difficulty with eating, reflux, and pain) and six single items (trouble swallowing saliva, choking at swallowing, dry mouth, trouble with tasting, coughing, and talking). The Chinese version of the scale is reliable and acceptable to measure the health-related QOL of patients with esophageal cancer in China [41]. In addition to QOL, the patients’ body weight and physical fitness measured by the 6MWD were assessed by medical doctors. Psychological status was measured using the Chinese versions of Patient Health Questionnaire-9 (PHQ-9) for depressive symptoms, General Anxiety Disorder-7 (GAD-7) for anxiety, and Perceived Stress Scale-10 (PSS-10) for stress. All these measures have high validity and reliability in Chinese populations [42-44].

### Data Management and Statistical Analyses

Descriptive analyses of the sociodemographic characteristics and health outcomes were conducted. Means and SDs were used to describe normally distributed continuous variables, while medians and IQRs were used for continuous variables that were not normally distributed, and proportions were used for categorical variables. Pre-post comparisons of outcomes between baseline and the 1-month follow-up and between baseline and the 3-month follow-up were conducted. Paired Student *t* tests were used for normally distributed continuous variables, Wilcoxon signed-rank tests were used for nonnormally distributed variables, and chi-square tests were used for categorical variables. The rates of depressive symptoms and anxiety in the participants were calculated using the cutoff scores of at least 10 and 7 for PHQ-9 and GAD-7, respectively [45,46]. The analyses were performed using SAS version 9.4 (SAS Institute, Inc).

## Results

### Patient Engagement

In total, 95% (19/20) of the participants used the online program. Moreover, participants used the online program for an average of 71 minutes in total during the study period. Participants viewed on average 84% (3.38/4) of the online video intervention content and completed on average 14% (3.20/23) and 34% (9.44/28) of the online audio and article content, respectively. Participants completed on average 63% (5.01/8), 100.00% (1/1), and 24% (10.89/46) of the online nutrition, physical exercise, and psychological intervention content, respectively. There was no serious adverse event in any of the participants.

### Patients’ Characteristics

Participants’ sociodemographic and clinical characteristics are summarized in Table 2. The mean age of the participants was 62.2 years (SD 7.1 years). The majority (18/20, 90%) of the participants were male, and more than half lived in rural regions (12/20, 60%) and did not complete high school (11/20, 55%). Most participants (13/20, 65%) had not received neoadjuvant chemotherapy or radiotherapy, and half (10/20, 50%) had a tumor in the middle thoracic area.

**Table 2:** Demographic and clinical characteristics of the study participants.

Characteristic		Participants (N=20), mean (SD) or n (%)
Age, years		62.20 (7.10)
**Gender**		
	Male	18 (90)
	Female	2 (10)
**Region**		
	City	8 (40)
	Rural	12 (60)
**Educational status**		
	Less than high school	11 (55)
	High school or greater	9 (45)
**Marital status**		
	Married	20 (100)
	Unmarried	0 (0)
**Parenting**		
	Yes	19 (95)
	No	1 (5)
**Occupation**		
	Retired	12 (60)
	Employed	8 (40)
**Family monthly income**, yuan (¥)^a^		
	<3000	11 (55)
	≥3000	9 (45)
Smoking		17 (85)
Drinking		9 (45)
Kungfu tea drinking		10 (50)
Regular exercise		10 (50)
Cancer history		9 (45)
**Neoadjuvant chemotherapy/radiotherapy**		
	Not performed	13 (65)
	Performed	7 (35)
**Tumor location**		
	Upper thoracic area	4 (20)
	Middle thoracic area	10 (50)
	Lower thoracic area	6 (30)
**Pathological stage**		
	I	4 (20)
	II	6 (30)
	III	9 (45)

^a^¥1 = US $0.14.

### Outcomes of the CIMmH Program

Table 3 presents the scores of all outcome variables at baseline, the 1-month follow-up, and the 3-month follow-up.

#### Quality of Life

Overall QOL decreased significantly (*P*=.02) from baseline to the 1-month follow-up, and symptoms, including fatigue (*P*<.001), pain (*P*=.004), dyspnea (*P*<.001), difficulty with eating (*P*=.005), trouble with coughing (*P*=.02), trouble with talking (*P*<.001), and reflux (*P*=.04), were aggravated. From baseline to the 3-month follow-up, most of the QOL dimensions returned to the preoperative level, except for pain (*P*=.02), diarrhea (*P*=.04), difficulty with eating (*P*=.03), and trouble with talking (*P*=.003). Compared with baseline findings, the symptom of dry mouth was significantly alleviated at the 3-month follow-up (*P*=.04).

#### Nutrition Status

Participants’ nutrition status worsened after esophagectomy. Analyses of pre-post changes showed a significant decrease in weight (*P*<.001) and BMI (*P*=.02) from baseline to the 1-month follow-up. Similarly, there was a significant decrease in weight (*P*<.001) and BMI (*P*=.02) from baseline to the 3-month follow-up.

#### 6-Minute Walk Distance

There was no significant change in the 6MWD between baseline and the 1-month follow-up (*P*=.22) or between baseline and the 3-month follow-up (*P*=.52).

#### Psychological Outcomes

There was a significant increase in depressive symptoms from baseline to the 1-month follow-up (*P<*.001). From baseline to the 3-month follow-up, the change in depressive symptoms was not statistically significant (*P*=.08). There were also no significant changes in anxiety (from baseline to the 1-month follow-up: *P*=.48; from baseline to the 3-month follow-up: *P*=.59) and perceived stress levels (from baseline to the 1-month follow-up: *P*=.06; from baseline to the 3-month follow-up: *P*=.78) throughout the study. Based on the cutoff scores of the measures, 15% (3/20) of patients developed depressive symptoms 1 month after surgery, while 6% (1/16) still had depressive symptoms at the 3-month follow-up. With regard to anxiety, 20% (4/20) of patients had anxiety at baseline, 20% (4/20) had it at the 1-month follow-up, and 12% (2/16) still had it at the 3-month follow-up.

**Table 3:** Results of the outcome variables.

Outcome variables				Baseline score or value^a^ (N=20)	1-month follow-up score or value^a^ (N=20)	3-month follow-up score or value^a^ (N=16)
**Quality of life**						
	**EORTC-QLQ-C30** ^b^					
		Overall quality of life scale^c^		76.70 (17.40)	65.40 (16.10)^d^	69.80 (12.10)
		**Functioning scale** ^e^				
			Physical functioning	93.70 (12.30)	84.00 (17.30)	90.80 (8.20)
			Role functioning	80.00 (29.80)	72.50 (23.70)	74.00 (20.60)
			Emotional Functioning	86.70 (13.20)	76.70 (26.00)	85.40 (20.70)
			Cognitive functioning	94.20 (11.80)	90.00 (18.10)	92.70 (11.40)
			Social functioning	71.70 (25.80)	70.80 (22.90)	65.60 (25.90)
		**General symptom scale** ^f^				
			Fatigue	10.00 (12.30)	36.10 (19.70)^g^	20.80 (23.10)
			Nausea and vomiting scale	5.00 (11.60)	12.50 (22.90)	5.20 (15.90)
			Pain	3.30 (6.50)	15.80 (18.20)^g^	12.50 (15.70)^d^
			Dyspnea	3.30 (14.20)	25.00 (17.40)^g^	20.80 (27.70)
			Insomnia	23.30 (32.70)	30.00 (32.40)	22.90 (35.60)
			Appetite loss	6.70 (22.10)	20.00 (26.00)	20.80 (27.70)
			Constipation	11.70 (21.30)	13.30 (21.60)	16.70 (28.00)
			Diarrhea	6.70 (13.00)	13.30 (19.00)	12.50 (15.70)
			Financial difficulties	36.70 (28.90)	28.30 (33.00)	29.20 (25.20)
	**EORTC-QLQ-OES18** ^h^					
		**General functional scale** ^e^				
			Dysphagia	68.30 (33.80)	62.80 (27.50)	68.10 (29.60)
		**General symptom scale** ^f^				
			Trouble swallowing saliva	36.70 (44.70)	31.70 (39.10)	26.70 (37.60)
			Choked when swallowing	16.70 (28.20)	23.30 (25.40)	28.90 (33.10)
			Eating	6.70 (14.70)	25.80 (18.20)^g^	21.70 (16.40)^d^
			Dry mouth	20.00 (28.00)	23.30 (25.40)	6.70 (12.90)^d^
			Trouble with taste	5.00 (21.30)	11.70 (23.60)	2.20 (8.10)
			Trouble with coughing	11.70 (18.60)	38.30 (33.00)^d^	15.60 (20.00)
			Trouble talking	0.00 (0.00)	43.30 (29.30)^g^	17.80 (20.00)^g^
			Reflux	5.00 (14.60)	23.30 (26.90)^d^	25.60 (31.70)
			Pain	8.30 (14.10)	10.60 (12.10)	10.40 (13.30)
**Nutrition status**						
	Weight (kg)			60.00 (8.70)	56.30 (7.80)^g^	55.00 (8.00)^g^
	BMI (kg/m^2^)			21.50 (3.30)	20.50 (2.60)^d^	20.00 (2.60)^d^
**Physical fitness**						
	6MWD^i^ (m)			506 (330.00-558.00)	469 (276.00-612.00)	486 (343.00-682.00)
	6MWD change^j^			N/A^k^	0.95 (0.67-1.43)	1.03 (0.83-1.24)
**Psychological measures**						
	PHQ-9^l^			1.11 (1.33)	5.00 (4.61)^d^	2.81 (3.56)
	GAD-7^m^			3.50 (4.16)	4.20 (4.54)	2.65 (3.52)
	PSS-10^n^			10.30 (4.54)	12.60 (6.61)	10.65 (7.03)
	Depressed, n (%)			0 (0)	3 (15)	1 (6)
	Anxiety, n (%)			4 (20)	4 (20)	2 (12)

^a^Data are presented as mean (SD), n (%), or median (range).

^b^EORTC-QLQ-C30: European Organization for Research and Treatment of Cancer-Quality of life Question-Core-30.

^c^Higher scores indicate better health.

^d^*P*<.05.

^e^Higher scores indicate better function.

^f^Higher scores indicate worse symptoms.

^g^*P*<.01.

^h^EORTC-QLQ-OES-18: European Organization for Research and Treatment of Cancer-Quality of life Question-Oesophageal Cancer Module-18.

^i^6MWD: 6-minute walk distance.

^j^6MWD change was calculated using follow-up 6MWD values divided by baseline 6MWD values.

^k^N/A: not applicable.

^l^PHQ-9: Patient Health Questionnaire-9.

^m^GAD-7: General Anxiety Disorder-7.

^n^PSS-10: Perceived Stress Scale-10.

### Qualitative Feedback

At the end of the study, participants were asked about their experiences of the intervention through the WeChat platform. Of the 20 participants, 18 (90%) reported that they were satisfied with the intervention program. Some suggested that the intervention interface and content could be designed in a more interesting and attractive way. One patient suggested that more intervention programs should be delivered in video format. Eight patients reported that they would like to receive more information about effective strategies to cope with postoperative complications.

## Discussion

### Principal Results

To the best of our knowledge, this prospective pilot study is the first attempt to develop and test the feasibility of an mHealth-based comprehensive intervention with nutrition, exercise, and psychological support through an online platform to help promote the ERAS program for patients with esophageal cancer.

For patients with cancer, ERAS is critical and challenging, as they experience both physical and mental complications. Tailor-made comprehensive interventions are needed for patients with specific cancers, as different types of cancers have different needs. Patients with esophageal cancer, for example, need special attention for nutrition intake and rehabilitation of respiratory movement after esophagectomy. The CIMmH is a comprehensive intervention addressing poor nutrition, physical inactivity, and intensified mental health symptoms within a single program for patients with esophageal cancer after surgery. The findings from this study indicate that the CIMmH is feasible and safe with no serious adverse effects for patients. The relevant decrease in overall QOL and increases in symptoms like fatigue, pain, dyspnea, difficulty with eating, trouble with coughing, trouble with talking, and reflux at the 1-month follow-up were expected, as patients were still in the recovery period after the surgery and were using feeding tubes. The results indicated that at the 3-month follow-up, except for pain, difficulty with eating, dry mouth, and trouble with talking, most of the QOL measures returned to the levels at the preoperative stage, indicating that recovery in these dimensions occurred 3 months after surgery. Compared with this study, previous studies that used traditional postoperative rehabilitation programs reported greater decreases in most functional dimensions of QOL and more serious deterioration of symptoms at 1 month and 3 months after surgery [47-49]. The CIMmH facilitated more comprehensive recovery for patients undergoing esophagectomy by restoring their declining functions faster and easing the symptoms caused by surgical injury. Nevertheless, patients still reported more problems with talking, coughing, and eating at 3 months after the surgery compared with the preoperative stage. These issues might stem from neurological injury during surgery, requiring a longer recovery time. These findings therefore highlight the need for routine evaluation and assessment of the postoperative functional status in patients. Careful consideration of the effects of possible complications on functional outcomes after surgery is thus needed when providing counseling services to patients before they make their decisions to undergo surgery and to plan rehabilitation.

As surgical injury often worsens the nutritional status of patients with esophagectomy, decreases in body weight and BMI are expected and have been well documented [8,50]. However, the drop in BMI was smaller in this study (1.0 and 1.5 at the 1- and 3-month follow-ups, respectively) than in a previous report (1.9 and 2.3 at the 1- and 3-month follow-ups, respectively) [50]. The minor drop in nutritional measures in this study indicates improved nutritional outcomes compared with the previous study [50].

Results of the 6MWD test at the 3-month follow-up demonstrated physical status comparable to that at baseline, indicating the effects of the CIMmH with regard to buffering the deterioration of physical fitness in patients after esophagectomy. Previous studies involving traditional postoperative rehabilitation showed a greater decrease in the 6MWD in the third month after surgery when compared with the finding in this study [51,52]. Lastly, with respect to psychological outcomes, depressive symptoms greatly increased at the 1-month follow-up, while anxiety and stress did not change greatly across all time points. Increased scores for depressive symptoms were expected as patients were greatly affected by surgical injuries at 1 month and were experiencing difficulties in eating, speaking, and even breathing in some cases. Nevertheless, the rates of depressive symptoms and anxiety in the present sample at 3 months after surgery were 6% (1/16) and 12% (2/16), respectively, whereas in a previous study, more than 40% of patients had depressive symptoms and anxiety after surgery [53], showing the potential of the CIMmH to support patients in coping with their conditions.

In this study, the mHealth system yielded a unique opportunity to provide much needed postsurgical care for the included patients. The functions of automatic monitoring, timely interventions, and feedback through the online mHealth system helped improve intervention adherence, as 80% (16/20) of participants completed the intervention. Although no formal qualitative data were collected in this study, some participants reported that they liked the video talks given by the medical doctors on how to take care of themselves and found the information on nutrition, exercise, and symptom management useful and helpful. In addition, professionals in the hospital reflected that the comprehensive intervention model of combining online (mHealth) and offline (face-to-face) services in this study was cost-effective and easier to incorporate into existing clinical practice and health care services, as less professional time was required and patients could receive tailor-made and timely interventions at home [54]. Given the large number of patients with esophageal cancer in low- and middle-income countries, the CIMmH has the potential to be a feasible cost-effective therapeutic option for improving postsurgery recovery in patients with esophageal cancer after esophagectomy.

Patient engagement data indicated that online intervention content in video format was more popular than audio or written materials. One possible reason might be that most participants in this study were elderly people who might have found it easier to understand vivid videos compared with audio content and articles. Future interventions may consider using more intervention materials in video format. Moreover, the completed proportions of the online nutrition and exercise intervention content were much higher than the completed proportion of the psychological intervention content. One possible explanation could be that the mental health status of the participants at baseline was better than that during follow-ups, so it was very likely that participants paid more attention to coping with postoperative complications than mental health–related issues. Another reason might be that there was insufficient emphasis on the importance of mental health at the beginning of the program. Mental health is an important problem in patients with esophageal cancer, as many of these patients experience depressive symptoms and anxiety after surgery [53,55]. Future interventions should emphasize the importance of mental health issues and educate patients to pay attention to their mental wellness after surgery.

### Implications

The CIMmH has yielded a unique opportunity to provide much needed postsurgical care in patients with esophageal cancer for the likely improvement of postoperative nutrition and the physical and mental status. This pilot study has shown that the CIMmH approach is a feasible and well-received option for ERAS in patients with esophageal cancer. Experiences of the CIMmH pilot study may help future development of a large randomized controlled trial or similar programs for patients with esophageal cancer or other cancers, especially those who are not able to visit the hospital frequently or who reside in rural areas. For example, patient adherence to the program needs to be enhanced in future interventions for better treatment effects, especially in the component of psychological intervention. More intervention programs should be delivered in video format, as video talks by medical doctors on patient self-care are particularly well received by patients.

### Limitations

Despite the positive outcomes, there were several limitations in this study. First, the sample size was small and there was no comparison group; thus, caution is needed to avoid over interpreting the findings. Second, some outcome data were missing owing to the drop-out of several patients at follow-up assessments. Third, effects of the CIMmH might be influenced by adherence and complications after surgery, which differ from patient to patient. Future studies should adopt a larger sample size and preferably use a randomized controlled trial design. The subjective experience of the participants should also be explored by collecting qualitative feedback throughout the study.

### Conclusions

The CIMmH is the first mHealth-based comprehensive intervention developed and tested in patients with esophageal cancer after esophagectomy. Our results show that the CIMmH is usable, feasible, and safe among patients with esophageal cancer after surgery in China. Future studies with a more rigorous design and larger samples are needed to establish efficacy in patients with esophageal cancer and those with other types of cancers.

## Abbreviations

6MWD: 6-minute walk distance
CIMmH: comprehensive intervention model using the mobile health system
EORTC-QLQ-C30: European Organization for Research and Treatment of Cancer-Quality of life Question-Core-30
EORTC-QLQ-OES-18: European Organization for Research and Treatment of Cancer-Quality of life Question-Oesophageal Cancer Module-18
ERAS: enhanced recovery after surgery
GAD-7: General Anxiety Disorder-7
MBCR: mindfulness-based cancer recovery
mHealth: mobile health
ONS: oral nutrition supply
PEN: partial enteral nutrition
PHQ-9: Patient Health Questionnaire-9
POD: postoperative day
PSS-10: Perceived Stress Scale-10
QOL: quality of life
TEN: total enteral nutrition
